# Structural Insights into the Rrp4 Subunit from the Crystal Structure of the *Thermoplasma acidophilum* Exosome

**DOI:** 10.3390/biom14060621

**Published:** 2024-05-24

**Authors:** Seonha Park, Hyun Sook Kim, Kyuhyeon Bang, Ahreum Han, Byeongmin Shin, Minjeong Seo, Sulhee Kim, Kwang Yeon Hwang

**Affiliations:** 1 Department of Biotechnology, College of Life Sciences and Biotechnology, Korea University, Seoul 02841, Republic of Korea; psh3810@korea.ac.kr (S.P.); k970329@hanmail.net (H.S.K.); qptmh360@naver.com (K.B.); yaringong@ibs.re.kr (A.H.); kingboom2@naver.com (B.S.); smj6247@korea.ac.kr (M.S.); 2 Institute of Bioresources, Korea University, Seoul 02841, Republic of Korea; 3 Korea BioDefense Research Institute, Korea University, Seoul 02841, Republic of Korea; sulhee@korea.ac.kr

**Keywords:** exosome, RNase, PNPase, RNA processing, quality control

## Abstract

The exosome multiprotein complex plays a critical role in RNA processing and degradation. This system governs the regulation of mRNA quality, degradation in the cytoplasm, the processing of short noncoding RNA, and the breakdown of RNA fragments. We determined two crystal structures of exosome components from *Thermoplasma acidophilum* (*Taci*): one with a resolution of 2.3 Å that reveals the central components (*Taci*Rrp41 and *Taci*Rrp42), and another with a resolution of 3.5 Å that displays the whole exosome (*Taci*Rrp41, *Taci*Rrp42, and *Taci*Rrp4). The fundamental exosome structure revealed the presence of a heterodimeric complex consisting of *Taci*Rrp41 and *Taci*Rrp42. The structure comprises nine subunits, with *Taci*Rrp41 and *Taci*Rrp42 arranged in a circular configuration, while *Taci*Rrp4 is located at the apex. The RNA degradation capabilities of the *Taci*Rrp4:41:42 complex were verified by RNA degradation assays, consistent with prior findings in other archaeal exosomes. The resemblance between archaeal exosomes and bacterial PNPase suggests a common mechanism for RNA degradation. Despite sharing comparable topologies, the surface charge distributions of *Taci*Rrp4 and other archaea structures are surprisingly distinct. Different RNA breakdown substrates may be responsible for this variation. These newfound structural findings enhance our comprehension of RNA processing and degradation in biological systems.

## 1. Introduction

The exosome is a multiprotein complex that participates in the processing and degradation of RNA molecules. It was initially found in *Saccharomyces cerevisiae* as a 3′→5′ exoribonuclease [1]. Later, exosomes were identified in diverse organisms, including humans [2], plants [3], and archaea [4]. Several studies have linked exosomes to the quality control of messenger RNA (mRNA) [5], the turnover of cytosolic messenger RNA (mRNA), and the processing of short noncoding RNA (sncRNA) [6]. In addition, the exosome is associated with RNA fragment breakdown [7]. In both archaea and eukaryotes, the exosome is therefore essential for RNA processing.

In general, eukaryotic exosomes are composed of nine subunits, divided into RNase and RNA-binding subunits. The yeast core exosome contains, for instance, six RNase-PH-like subunits (Rrp41, Rrp42, Rrp43, Rrp45, Rrp46, and Mtr3) and three RNA-binding proteins (Csl4, Rrp4, and Rrp40) with an S1 or a K homology (KH) domain, which is a protein domain that was first identified in the human heterogeneous nuclear ribonucleoprotein K (hnRNP) [8,9,10,11]. According to structural investigations, six RNase-PH-like subunits form a hexameric ring that interacts with three RNA-binding subunits. Archaeal exosomes, unlike those of eukaryotes, contain only two RNase-PH-like subunits, Rrp41 and Rrp42. These subunits form a hexameric ring where Rrp41 and Rrp42 exist as heterodimers. Archaeal exosomes also contain RNA-binding proteins, Csl4 or Rrp4, indicating that the fundamental structure of exosomes is species-consistent.

The RNA-binding activities of exosomes might be physically confirmed from the structure of bacterial polynucleotide phosphorylase (PNPase) [12,13]. PNPase is the primary eubacterial mRNA degradation enzyme. In a single polypeptide, bacterial PNPase comprises two RNase PH domains, a KH domain, and an S1 domain. The three S1 and KH domains are positioned on one side of a hexameric ring formed of six RNase PH domains. Although the detailed subunits are different, it has been argued that the roles of archaeal exosomes and bacterial PNPase may be similar due to their structural homology. Several exosome structures have been revealed in archaea from *Arhaeoglobus fulgidus* (*Aful*) [14], *Methanothermobacter thermautotrophicus* [15], *Pyrococcus abyssi* [16], and *Sulfolobus solfataricus* (*Ssol*) [17,18]. These structures led researchers to discover the apo or RNA-bound forms of exosomes, validating the fundamental framework for RNA binding [19]. Particularly, the investigation of the exosome of *Ssol* revealed that only the Rrp41 subunit possesses catalytic activity, but its activation needs a connection with Rrp42 [18]. Here, to clearly understand this ability, we determined the two archaeal exosome structures of *Thermoplasma acidophilum* (*Taci*). The *Taci*Rrp41 and *Taci*Rrp42 structural component revealed a heterodimeric complex structure of the exosome core at 2.3 Å. In addition, the structure of the whole exosome (*Taci*Rrp41, *Taci*Rrp42, *Taci*Rrp4) at 3.5 Å revealed a ring structure similar to that of bacterial PNPase. Its complex structure consists of nine subunits, with *Taci*Rrp41 and *Taci*Rrp42 trimerizing to form a ring structure and *Taci*Rrp4 positioned at the complex’s apex. An RNA degradation assay was conducted using individual (*Taci*Rrp4 or *Taci*Rrp42) and complexed (*Taci*Rrp41:42 or *Taci*Rrp4:41:42) to identify the core subunit responsible for RNA degradation ability. The optimal degradation ability was achieved when all three complexes were assembled to form a unified structure. The diminished RNA degradation capability in subunits *Taci*Rrp4 and *Taci*Rrp42 may indicate that these subunits are not crucial for the observed RNA degradation process. Based on structural and biochemical analyses, we will describe the main differences between PNPase and archaeal exosome structures.

## 2. Materials and Methods

### 2.1. Cloning and Protein Expression

The cDNA encoding the Rrp41 of the *Thermoplasma acidophilum* exosome was amplified using the primer pair forward: 5′-CGGGATCCGATGAAAAGGATGGAAGCC-3′, and reverse: 5′-ACGCGTCGACTCACTCACCCTCTCCG-3′. The subunit for *Taci*Rrp42 (the truncated Rrp42 corresponding to the core domain composed of 20–240 residues) was also amplified using the primer pair forward: 5′-GGAATTCCATATGATGAAGGGCGG GAAGA-3′ and reverse: 5′-CGCCTCGAGTCACCTGAAGTATTTTTCC-3′. The amplified *Taci*Rrp41 was cloned into the pET22b vector, and *Taci*Rrp42 was cloned into the pET28a vector. Also, both *Taci*Rrp41 and *Taci*Rrp42 subunits were cloned into the pETDuet-1 vector (Novagen) using DNA fragments that had been digested with the restriction enzymes BamHI, SalI, NdeI, and XhoI. The subunit for *Taci*Rrp4 was cloned into the pET22b vector. The *Taci*Rrp41, *Taci*Rrp42, and *Taci*Rrp41:42 recombinant plasmids were transformed into *Escherichia coli* BL21 (DE3), and the *Taci*Rrp4 plasmid was transformed into Rosetta cells. These four types of cells were grown individually at 310 K in Luria broth (LB) medium until an OD600 of 0.8 was reached. The expression of each protein was induced with 0.5 mM IPTG (isopropyl-β-D-thiogalactopyranoside) at 291 K for 18 h. The cells were harvested by centrifugation at 277 K at 12,000× *g* for 20 min.

### 2.2. Protein Production and Crystallization

Harvested cells of each subunit (Rrp4, Rrp41, Rrp42, Rrp41:42) were resuspended in buffer A (Tris 30 mM, NaCl 150 mM, pH 7.6) and disrupted by sonication. After centrifugation, the supernatant was purified by Ni-NTA affinity chromatography using a HisTrap^TM^ HP column. A linear gradient of imidazole concentration (0.02–0.5 M) was used to elute the proteins. The fractions were confirmed by SDS-PAGE and the protein was further purified by gel-filtration chromatography on a Superdex 200 column in the final buffer (Tris 30 mM, NaCl 150 mM, pH 7.6). This method was used to gain single-subunit proteins and binary complex proteins. To obtain the ternary complex, the *Taci*Rrp41:42 and *Taci*Rrp4 proteins were incubated for an hour at room temperature, followed by additional gel-filtration chromatography to separate the ternary complex. The proteins were concentrated using Amicon ultracentrifugal filters. Binary (*Taci*Rrp41:42) and ternary (*Taci*Rrp4:41:42) cocrystals were obtained by the hanging drop vapor diffusion method by combining 1 μL of the protein and 1 μL of the reservoir solution (0.08 M sodium acetate trihydrate, 1.6 M ammonium sulfate, and 20% glycerol, pH 4.9). The crystals grew within 5 days at 22 °C.

### 2.3. Data Collection and Structure Determination

Diffraction data were collected using the beamline at the Pohang Accelerator Laboratory. The data set for the *Taci*Rrp41:42 complex crystal was collected at a 2.3 Å resolution, and the complete exosome structure (*Taci*Rrp4:41:42) was collected at a 3.5 Å resolution. The structure was determined by molecular replacement using the structure of *Pyrococcus abyssi* (PDB ID: 2PNZ) as a search model. Further model building was conducted using Coot, and refinement was performed using REFMAC and PHENIX. The final model for *Taci*Rrp41:42 had R values of R_work_ = 0.20 and R_free_ = 0.24 at a 2.3 Å resolution, and the exosome structure *Taci* Rrp4:41:42 had R values of R_work_ = 0.23 and R_free_ = 0.27 at a 3.5 Å resolution. The statistics for the detailed data collection and structure refinement are provided in Table 1.

### 2.4. RNA Assay

RNA degradation assay was conducted with a ^32^P-5′-labeled 30-mer poly(A) oligoribonucleotide. A total of 100 pmol of RNA was labeled with 40 μCi γ-[^32^P] ATP and T4 polynucleotide kinase (NEB) for 50 min at 37 °C, and unincorporated ribonucleotides were eliminated using MicroSpin G-25 columns (Amersham). A total of 100 pmol of each protein was incubated with the RNA substrate in a 50 μL buffer containing 20 mM Tris-HCl (pH 7.8), 60 mM KCl, 10 mM MgCl_2_, 10% glycerol, 2 mM DTT, 0.1 mM EDTA, and 10 mM NaH_2_PO_4_ for 10 min or 20 min at 50 °C. DEPC water was added instead of exosome protein for the control band. The reaction was stopped by adding 1 volume of staining solution to the reaction mixture. The reaction products were resolved on a 15% (*v*/*v*) TBE–urea polyacrylamide gel and were analyzed by phosphorimaging (Amersham Typhoon).

### 2.5. Microscale Thermophoresis (MST) Measurement

MST measurement was carried out to identify molecular interactions between exosomal subunits. *Taci*Rrp42, as the target protein, was labeled fluorescently using a His-tag Labeling Kit (The Monolith His-Tag Labeling Kit RED-tris-NTA 2nd generation: Nanotemper technologies) at a 50 nM concentration. *Taci*Rrp41 was used as a ligand at concentrations ranging from 550 μM to 16.6 nM, prepared using sixteen serial dilutions. The ligand *Taci*Rrp4 was also prepared in the same way, with concentrations ranging from 200 μM to 6.1 nM. Also, *Taci*Rrp41:42 was labeled fluorescently with the same labeling kit as the target protein. The ligand *Taci*Rrp4 was serially diluted to sixteen concentrations, starting at 200 μM and increasing to 6.1 nM. Target protein–ligand protein solutions with a ratio of 1:1 were incubated for 30 min. The measurements were conducted under conditions where the LED power and MST power were both at 40%. The affinity constant (Kd) was determined by curve fitting using MO Affinity Analysis software v.2.2.7 (Nanotemper). All measurements were conducted in triplicate.

### 2.6. Inflection Temperature (Ti) Measurement

Inflection temperature (Ti) was measured to compare the stability of exosomal subunits or complexes. Ti measurements were performed using a Tycho NT.6 system (Nanotemper). Proteins at a concentration of 1 mg/mL were prepared in PBS buffer, and loaded into capillaries (Nanotemper). While the samples were heated at a rate of 3 °C per minute between 35 °C and 95 °C, intrinsic fluorescence was detected at 330 nm and 350 nm. The ratio of fluorescence (350/330 nm) and the Ti were plotted by the Tycho NT.6 system.

## 3. Results

### 3.1. Overall Structures

**Figure 1:**
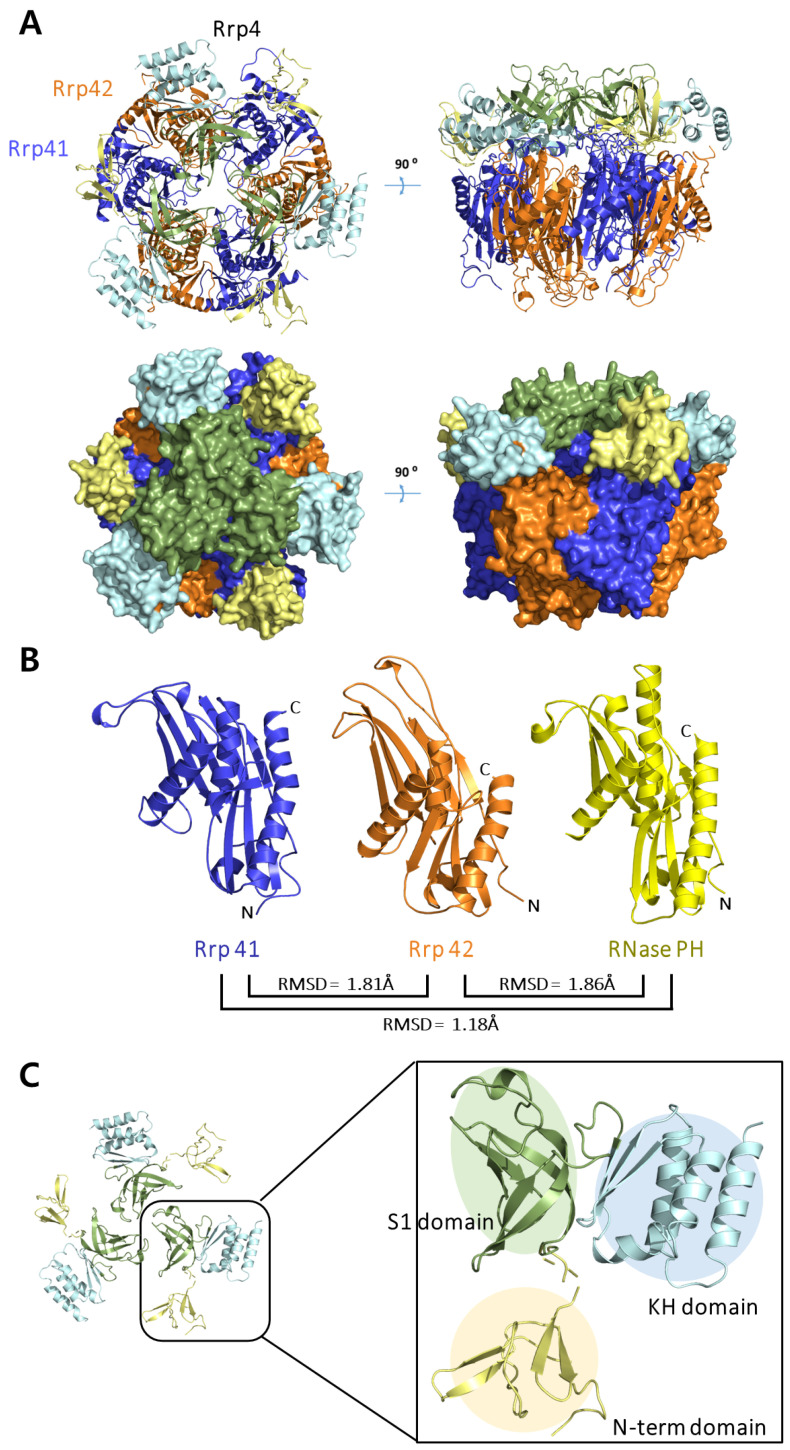
(**A**) Overall structure of the *Thermoplasma acidophilum* exosome. RNase PH subunits (*Taci*Rrp41 and *Taci*Rrp42) are displayed in blue and orange, respectively, and protein *Taci*Rrp4 is displayed in three colors based on the individual domains (N-term domain: pale yellow; S1 domain: green; and KH domain: sky blue). (**B**) Structural similarity of *Taci*Rrp41 (blue) and *Taci*Rrp42 (orange) with bacterial RNase PH (yellow). A total of 956 atoms were used in alignment with *Taci*Rrp41. A total of 949 atoms were used in alignment with *Taci*Rrp42. RNase PH is shown in a similar orientation to *Taci*Rrp41 and *Taci*Rrp42 subunits. *Taci*Rrp41 and RNase PH display the highest degree of similarity. (**C**) *Taci*Rrp4 consists of three domains: N-terminal domain (pale yellow), S1 domain (green), and KH domain (sky blue).

The crystal structure of *Taci*Rrp41:42 with a phosphate ion was solved at 2.3 Å and belonged to the P2_1_3 space group. The structure was refined to obtain an R_work_ of 0.20 and an R_free_ of 0.24. The full complex structure of *Taci*Rrp4:41:42 was solved at 3.5 Å and refined to obtain an R_work_ of 0.23 and an R_free_ of 0.27. The structure belonged to the P3_2_21 space group. The overall exosome structure of *Taci* consists of nine subunits, three *Taci*Rrp41 and *Taci*Rrp42 heterodimers forming a hexameric ring structure and capped on one side by three subunits of *Taci*Rrp4 (Figure 1A). Both *Taci*Rrp41 and *Taci*Rrp42 have an RNase-PH-like fold, with the characteristic of β-α-β layers of secondary structure elements. When compared with RNase PH of *Bacillus subtilis* [18], Rrp41 and Rrp42 of *Taci* showed r.m.s deviation of 1.18 Å (956 atoms used for alignment) and 1.86 Å (949 atoms used for alignment), respectively (Figure 1B). *Taci*Rrp4 consists of three domains: an N-term domain, a central S1 domain, and a C-terminal KH domain (Figure 1C). This is a common characteristic found in other archaeal exosomes, such as *Aful*Rrp4 and *Ssol*Rrp4 [14,20]. It was suggested that the flexibility of the S1/KH domains is related to the need to bind to various RNA substrates [13].

**Figure 2:**
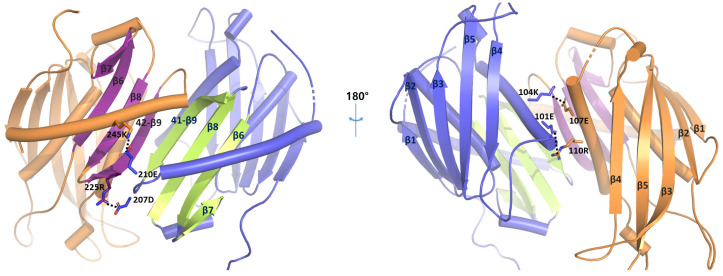
The arrangement of β strands when *Taci*Rrp41 and *Taci*Rrp42 combine. The helices are illustrated cylindrically. The 6–9th β strands of *Taci*Rrp41 are represented in lime color, while those of *Taci*Rrp42 are shown in purple. Salt bridges involved in dimerization stabilization are indicated by stick models. We analyzed the salt bridge within 3.5 Å at the dimer interface.

*Taci*Rrp41 and *Taci*Rrp42 work together within the exosome, ensuring efficient RNA degradation through their unique quaternary structure and active sites. Their collaboration allows precise control over RNA processing and quality control. Both subunits consist of four helices and nine β strands. When numbered sequentially, strands 1 to 5 and 6 to 9 form anti-parallel β sheets, with each sheet spatially separated by α helices. When the *Taci*Rrp41 and *Taci*Rrp42 subunits combine to form a complex, it was observed that the ninth β strands of each subunit aligned in an anti-parallel manner (Figure 2). Consequently, a total of eight β strands from each subunit were arranged in an anti-parallel structure. This arrangement is consistent with the known RNasePH domain structure [18]. Also, four salt bridges were found to contribute to the stabilization of dimerization. The three salt bridges were located between helices surrounding the eight β strands (210E^41^–245K^42^, 104K^41^–107E^42^, 101E^41^–110R^42^), while the remaining one was formed between a *Taci*Rrp42 β strand and a *Taci*Rrp41 loop (207D^41^–225R^42^) (the superscripts 41 and 42 stand for *Taci*Rrp41 and *Taci*Rrp42, respectively). These connections facilitate the protein’s maintenance of its hexamer shape, which consists of a trimer of dimers. Generally, *Taci*Rrp42 probably interacts with RNA and helps with substrate binding, whereas *Taci*Rrp41 makes sure the substrate has a strong affinity and stops RNA from being released too early during breakdown.

### 3.2. Active Site and Phosphate-Ion-Binding Site

**Figure 3:**
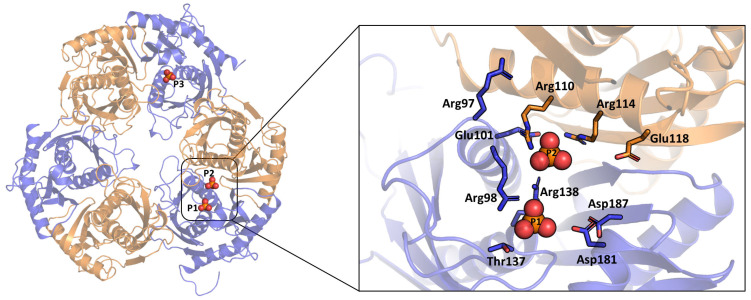
Detailed view from the bottom of the ring of the active site in the *Taci*Rrp41:42 complex. Inorganic phosphates are labeled P1, P2, and P3. The *Taci*Rrp41 subunit is colored in blue and *Taci*Rrp42 in orange.

The archaeal exosome comprises a heterohexameric processing chamber with three RNase-PH-like active sites. Subunits of either the Rrp4- or Csl4-type, which contain RNA-binding domains, top this chamber [21]. The RNase PH family includes the *Taci*Rrp41 subunit of the archaeal exosome. Three *Taci*Rrp41:42 dimers form the ring in archaea. The central chamber within the ring contains three phosphorolytic active sites located at the interface between *Taci*Rrp42 and *Taci*Rrp41 (Figure 3). We can infer that all three phosphate-binding sites share the same sites because the phosphate-binding sites of the two subunits (P1 and P3) are identical. Furthermore, many arginine residues from *Taci*Rrp41 and *Taci*Rrp42 are involved in forming a positively charged region around the inorganic phosphate.

**Figure 4:**
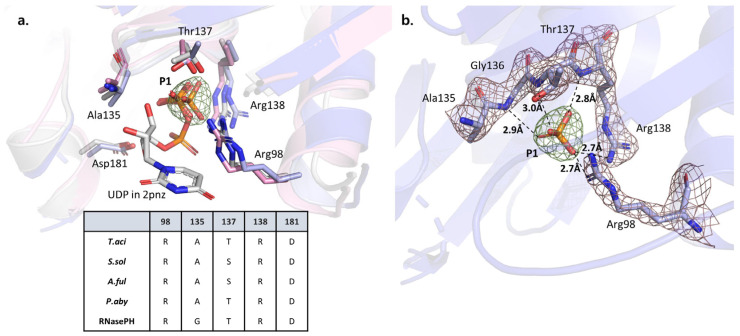
(**a**) A superimposed model of Rrp41 from *P. abyssi* (PDB ID: 2PNZ), *S. solfataricus* (PDB ID: 4BA1) and *T. acidophilum*. *P. aby* in white, *S. sol* in pink, and *T. aci* in blue. The sequence numbers written on residues are based on *T. aci* as the reference. The positions of the inorganic phosphate from *S. sol*, *T. aci*, and UDP (uridine-5′-diphosphate) from *P. aby* are very similar in the three different exosomes. A sequence alignment table of conserved residues surrounding the phosphate-binding site is provided below. (**b**) An electron density map of inorganic phosphate and contacting residues. Fo-Fc electron density map at 1 sigma is displayed in green for phosphate and brown for residues.

Previously determined crystal structures of *S. solfataricus* showed a phosphate-binding site in the *Ssol*Rrp41 subunit [16]. When superimposed on this structure, the position of the P1 phosphate coincided with the phosphate-binding site of *S. sol*. We confirmed the conservation of residues in the surrounding space. The only difference was the presence of serine at position 138 in *Ssol*Rrp41 in place of the threonine at position 137 in *Taci*Rrp41 threonine (Figure 4a). Furthermore, when overlaid with another previously elucidated exosome–UDP (uridine-5′-diphosphate)-binding structure from *P. abyssi*, the phosphate portion of UDP also aligned with that of *T. aci*. The conservation of the Pi-binding site position suggests that phosphorolytic exosome complexes share a mechanism of phosphate-dependent RNA degradation, as reported in archaeal exosomes as well as RNase PH and PNPase enzymes.

### 3.3. Structural Comparisons

**Figure 5:**
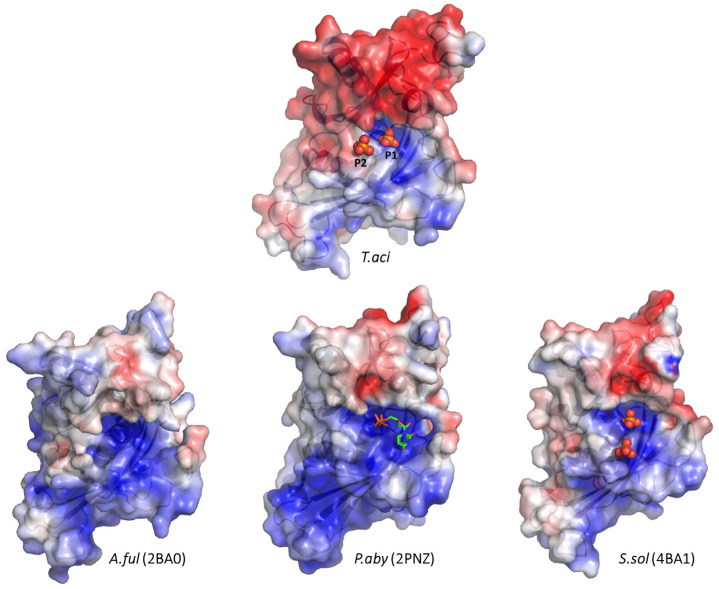
The electrostatic surface potential of archaeal exosomal Rrp41 subunits. *Aful*Rrp41 (PDB ID: 2BA0), *Paby*Rrp41 (PDB ID: 2PNZ), and *Ssol*Rrp41 (PDB ID: 4BA1) were used for comparisons. The range of electrostatic surface potential is shown from −5 kT/e (red color) to +5 kT/e (blue color). Inorganic phosphates are shown as red spheres, and UDP (uridine-5′-diphosphate) as a green stick model.

To compare them with other archaeal exosomes, we analyzed the surface structures of *Taci*Rrp41 and *Taci*Rrp42 individually. The surface morphology of Rrp41s exhibited similarities, but the electrostatic properties demonstrated noticeable differences (Figure 5). The r.m.s.d. values were 0.74 Å in *A*. *ful* (1228 atoms were used in the alignment), 0.84 Å in *P. aby* (1283 atoms were used in the alignment), and 0.81 Å in *S. sol* (1307 atoms were used in the alignment). In the previously described phosphate-binding sites of *S. sol* [14], one phosphate (P1) perfectly matched the phosphate-binding site of *T. aci*, while the other was in a distinct place.

**Figure 6:**
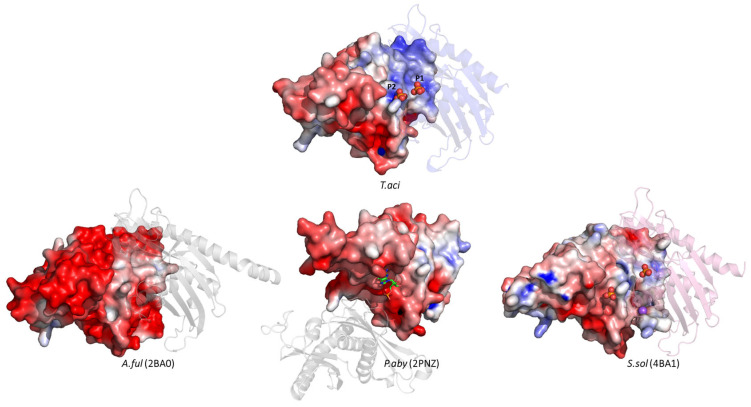
The electrostatic surface potential of archaeal exosomal Rrp42 subunits in a similar orientation. *Aful*Rrp42 (PDB ID: 2BA0), *Paby*Rrp42 (PDB ID: 2PNZ), and *Ssol*Rrp42 (PDB ID: 4BA1) were used for comparisons. The range of electrostatic surface potential is shown from −5 kT/e (red color) to +5 kT/e (blue color). The adjacent Rrp41 is depicted as a semi-transparent cartoon model. Inorganic phosphates are shown as red spheres, sodium ions as purple spheres, and 5GP (guanosine-5′-monophosphate) as a green stick model [16].

The surface model of Rrp42 also exhibited electrostatic differences (Figure 6). The r.m.s.d values with Rrp42 of *A.ful*, *P. aby*, and *S. sol* were 0.83 Å (1137 atoms were used in the alignment), 0.80 Å (1175 atoms were used in the alignment) and 0.84 Å (1066 atoms were used in the alignment), respectively. *Aful*Rrp42 exhibited a significant negative charge, represented by a prominent red color, while *Taci*Rrp42, in contrast, displayed both positive and negative charges. Both *Taci*Rrp42 and *Ssol*Rrp42 displayed positive charges in the proximity of their interaction with Rrp41.

**Figure 7:**
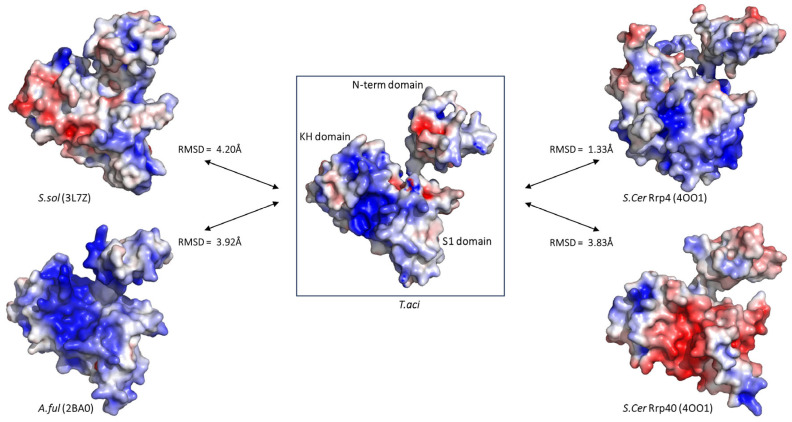
The electrostatic surface potential of archaeal exosomal Rrp4 subunits in a similar orientation. The range of electrostatic surface potential is shown from −5 kT/e (red color) to +5 kT/e (blue color). The Rrp4 of *T. aci* is indicated in a rectangular box. The Rrp4 of *S. sol* (PDB ID: 3L7Z), *A. ful* (PDB ID: 2BA0), and *S. cer* (PDB ID: 4OO1) and the Rrp40 of *S. Cer* (PDB ID: 4OO1) are used for comparison. The domains corresponding to each surface model in *Taci*Rrp4 are written within the box.

In contrast to Rrp41 or Rrp42, Rrp4 exhibited notable variations among many species. Interestingly, *Taci*Rrp4 displayed a morphology more similar to the exosome subunit of *Saccharomyces cerevisiae* (*Scer*), a kind of eukaryote. Eukaryotic exosomes include Rrp4 and Rrp40, which are homologous to archaeal Rrp4 [22]. Both subunits have S1- and KH RNA-binding domains. When superimposed with *Taci*Rrp4, *Scer*Rrp4 showed an r.m.s.d. value of 1.33 Å (677 atoms were used in the alignment) and *Scer*Rrp40 had an r.m.s.d. of 3.83 Å (1029 atoms were used in the alignment), while it displayed r.m.s.d. values of 4.20 Å (1118 atoms were used in the alignment) and 3.92 Å (1184 atoms were used in the alignment) when superimposed with *Ssol*Rrp4 and *Aful*Rrp4, respectively. However, the surface charge distribution differed among the several species. (Figure 7).

**Figure 8:**
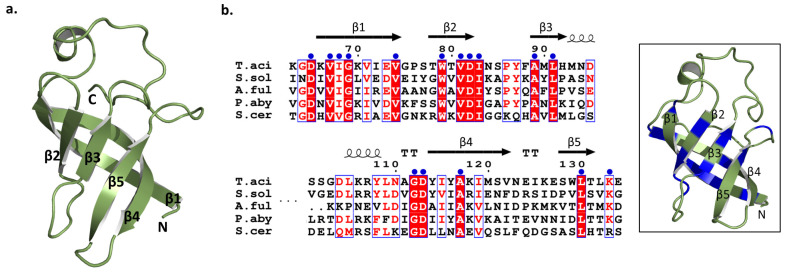
(**a**) The structure of the S1 domain in *Taci*Rrp4; (**b**) The sequence alignments of the S1 domain with other exosomes. The most conserved residues are marked with blue circles, and these regions are represented by the same blue color in the cartoon model on the right.

The archaeal exosome subunit Rrp4 possesses N-terminal, S1, and KH domains. The S1 domain and KH domain play crucial roles in binding to RNA. These domains are also seen in bacterial PNPase [23]. The S1 domain is composed of five-stranded anti-parallel β barrels (Figure 8a). Each β barrel is linked by four to six residue loops, except for strands 3 and 4. In *Taci*Rrp4, they have longer links of 19 residues and are positioned on the upper surface of the barrel structure. When comparing the S1 domain region with other archaeal exosomes and *S. cer*, it was observed that many residues were conserved. The majority of conserved sequences were grouped together either at the beginning or end of the β strand (Figure 8b). This implies that although the general structure of the S1 domain remains unchanged, alterations in other residues can result in differences in the binding capabilities among S1 domains. Not only is the S1 domain present in a significant number of RNA-associated proteins, but its configuration also closely resembles that of cold-shock proteins, suggesting the potential evolutionary origin of a common nucleic-acid-binding precursor protein [24].

**Figure 9:**
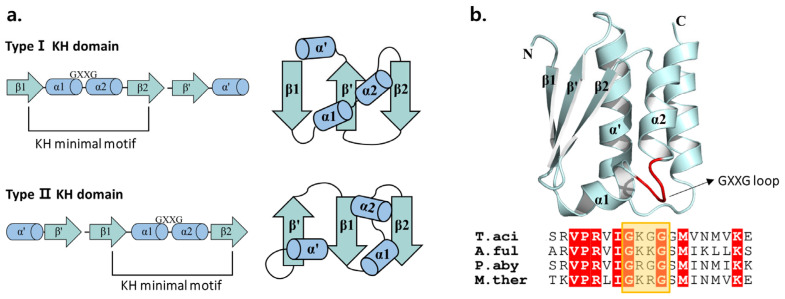
(**a**) Two types of KH domain; (**b**) Structure of KH domain in *Taci*Rrp4. GXXG loop is indicated by red color. Conserved sequences of GXXG loop among archaeal Rrp4 proteins are shown in yellow box at the bottom.

The KH domain is found in a wide range of nucleic-acid-binding proteins. The common function of the KH domain is the recognition of RNA or single-stranded DNA [25]. It has been noted that there are two different versions of the KH motif, referred to as type I and type Ⅱ KH folds [26]. The type I fold is commonly observed in eukaryotic proteins, while the type Ⅱ fold is predominantly found in prokaryotic proteins. The two types of KH folds share a similar basic framework but exhibit differences in their three-dimensional arrangements (Figure 9a). Type I KH domains feature a C-terminal extension consisting of a βα unit, and three-stranded β strands are arranged in an anti-parallel manner. Type Ⅱ KH domains harbor an N-terminal extension comprising an αβ unit, and two out of the three β strands are oriented in a parallel fashion.

In both types of KH domain, two α helices were linked by the GXXG loop. It is known that binding with nucleic acids occurs within a cleft formed between these helices and the GXXG loop, as well as other loops, through various protein-specific interactions [24]. The KH domain of *Taci*Rrp4 belonged to Type I (Figure 9b). When compared to other archaeal exosomes such as *A.ful*, and *P. aby*, there was a conserved sequence of VPRVIGXXG. Nevertheless, this loop was absent in both the *S. sol* and *S.cer* structures. From this, we can deduce that the loop sequence was preserved to a certain degree. The KH domain is not directly responsible for RNA degradation. However, it is believed to play a role in recognizing and distinguishing various forms of RNA, as well as determining the length of RNA [27].

**Figure 10:**
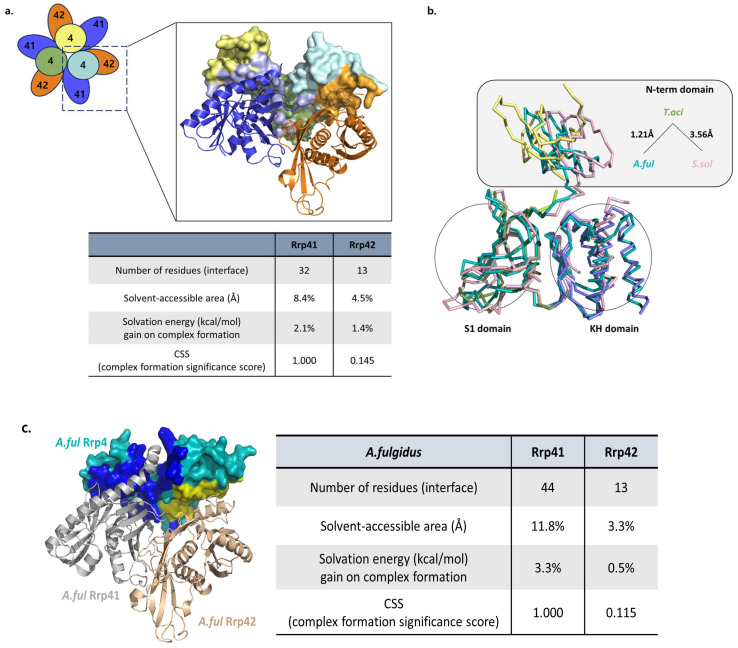
(**a**) The *Taci*Rrp4 interfaces interact with the *Taci*Rrp41 or *Taci*Rrp42 subunits. *Taci*Rrp4 is presented in the surface model. The light blue color on the surface shows residues interacting with *Taci*Rrp41, and the orange color on the surface shows residues interacting with *Taci*Rrp42. The number of residues, solvent-accessible area, solvation energy, and CSS (complex formation significance score) values are listed in the table. The CSS scores range from 0 to 1 as the interface relevance to complex formation increases. (**b**) Ribbon models overlaying the S1 and KH domains of three archaeal exosomes (*T. aci*, *A.ful*, *S. sol*). *T. aci* is represented in yellow, green, and blue according to the domains, while *A. ful* is shown in a teal color, and *S. sol* is depicted in pink. (**c**) The PISA interface data of *A. ful* subunits. *Aful*Rrp4 is presented in the surface model. The dark blue color on the surface shows residues interacting with *Aful*Rrp41, and the yellow color on the surface shows residues interacting with *Aful*Rrp42.

The N-terminal domain (NTD) of Rrp4 predominantly mediates the interactions with the hexameric core [17]. While it does not directly engage in RNA binding, it seems to have a vital function in binding with Rrp41 and Rrp42. Based on the PISA interface calculation program [28], it was observed that 32 residues of *Taci*Rrp41 were interacting with 29 residues of *Taci*Rrp4 (Figure 10a). Among the 29 residues, the majority (17 amino acids) were part of the NTD, while 11 were associated with the S1 domain, and only 1 was related to the KH domain. If interfaces associated with the S1 or KH domains are utilized to construct the substrate channel, a CSS (complex formation significance) score of 1 signifies the significance of the NTD in binding to the overall structure of the exosome. By comparison, *Taci*Rrp42 had 13 residues while *Taci*Rrp4 had 14 residues that formed interfaces, all of which were part of the KH domain. The CSS score was 0.145, indicating that this interface serves as a supplementary component in the development of complexes. *Aful*Rrp41 and *Aful*Rrp4 interacted with each other, with 44 residues of *Aful*Rrp41 interacting with 40 residues of *Aful*Rrp4. The CSS scoring for this interaction was 1.000. In addition, the 13 amino acid residues of *Aful*Rrp42 formed interactions with 17 amino acid residues of *Aful*Rrp4, leading to a CSS score of 0.115 (Figure 10c). Similar findings were also noted for *S. sol*. The CSS score was 0.520 at the interface of *Ssol*Rrp4-*Ssol*Rrp41 and 0.097 at *Ssol*Rrp4-*Ssol*Rrp42. The result of 0.097 indicates that it is not statistically significant for complex development. Based on the consistent outcomes of the interface calculations among archaeal exosomes, it can be deduced that the creation of an interface between Rrp4 and Rrp41 is essential for the production of the complex, with particular emphasis on the N-terminal domain of Rrp4. Furthermore, upon comparing the superimposed Rrp4s with the S1 and KH domains, it becomes evident that the three-dimensional positioning of the NTD differed across the structures (Figure 10b).

### 3.4. Bioassays

**Figure 11:**
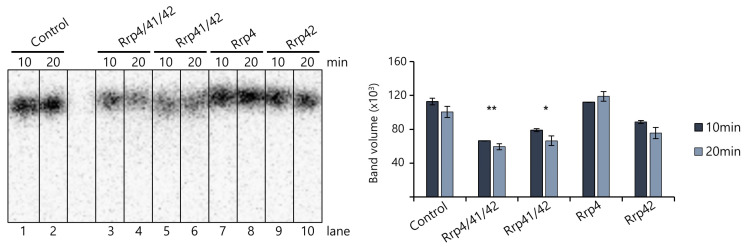
RNA degradation assay of *T. acidophilum* exosome subunits. *Taci*Rrp41:42 shows some degradation ability, but the ability is greatest when all three subunits (*Taci*Rrp4:41:42) are combined. The *Taci*Rrp4 subunit alone does not show RNase activity. In the control, DEPC water was added instead of exosome protein. The left panel shows a 15% polyacrylamide–urea gel. The graph representing the RNA band intensity of the left gel is displayed on the right. The number of * indicates the extent of degradation.

We performed various biochemical and biophysical characterizations to compare the biochemical functions of each exosome subunit from *T. acidophilum*. First, an RNase assay was conducted using ^32^P-5′-labeled 30-mer single-stranded poly(A) RNA. A band intensity observed following the addition of DEPC water was utilized as a reference. We then compared this intensity with the band intensity observed after the addition of exosomes to evaluate the exosomes’ ability to degrade. As a result, the *Taci*Rrp41:42 complex was capable of degrading a ^32^P-5′-labeled 30-mer single-stranded poly(A) RNA. However, this ability was significantly increased when taken together with the *Taci*Rrp4-capping protein (Figure 11). For *the Taci*Rrp4:41:42 complex, the band intensity was 58.9% compared to the negative control, whereas for the *Taci* Rrp41:42 complex, it showed a 69% band intensity compared to the negative control. *Taci*Rrp4 and *Taci*Rrp42 showed intensities of 99.3% and 78.6%, respectively, compared to the negative control. Because *Taci*Rrp4 and *Taci*Rrp42 subunits alone did not show striking RNase activity, it suggests that *Taci*Rrp41 plays a more important role in degrading RNA. In the *A. fulgidus* exosome, the activities were inhibited by the mutation in the active site in *Aful*Rrp41, whereas the mutation in *Aful*Rrp42 did not have an inhibiting effect [14].

**Figure 12:**
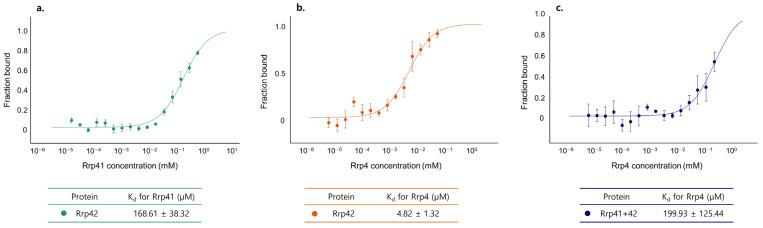
Binding affinity analysis of exosome subunits by MST experiments. (**a**) K_d_ values between *Taci*Rrp42 and *Taci*Rrp41. (**b**) K_d_ values between *Taci*Rrp42 and *Taci*Rrp4. (**c**) K_d_ values between *Taci*Rrp41:42 and *Taci*Rrp4.

Second, we measured the binding affinity of various exosome subunits using MST. Interestingly, the K_d_ of *Taci*Rrp42 with *Taci*Rrp4 is 4.82 µM, while that of the *Taci*Rrp41:42 complex is 199.9 µM. In addition, the K_d_ of *Taci*Rrp42 with *Taci*Rrp41 is 168.6 µM (Figure 12).

**Figure 13:**
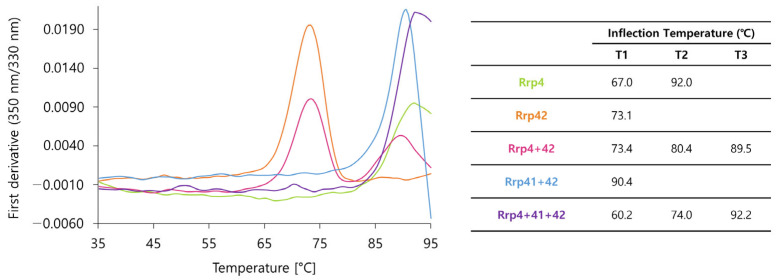
Tycho analysis of exosome subunits. The first derivatives of the ratio (350 nm/330 nm) are plotted. Inflection temperature (Ti) values are listed in the table.

Third, a thermal shift assay was conducted using Tycho NT.6 (Nanotemper) to compare the thermal stability of subunits. The results clearly show that each subunit is more stable when they form complexes than when they are alone. In particular, the T1 value of the *Taci*Rrp41:42 complex is 90.4 °C. This is higher than that of *Taci*Rrp42 alone (73.1 °C). In the case of the *Taci*Rrp4:41:42 complex, the fully unfolding temperature, T3, is 92.2 °C. This means this complex represents the highest stable form in the *Taci* exosome (Figure 13).

## 4. Discussion

**Figure 14:**
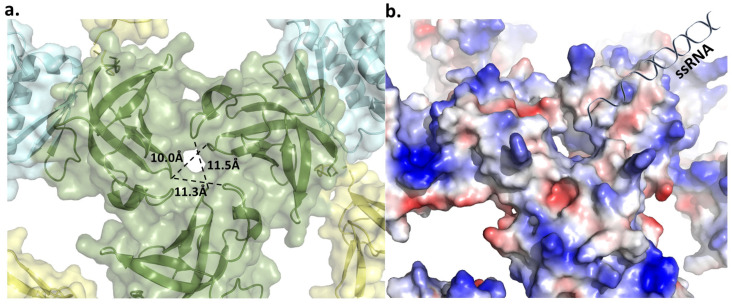
(**a**) The possible S1 pore of *Taci*Rrp4 and the distance between each S1 domain (**b**) A schematic diagram of ssRNA entering the S1 pore. According to the electrostatic model, the S1 pore is the entry point for RNA.

Based on previously described archaeal exosome structures, it has been observed that RNA enters through a specific opening called the ‘S1 pore’ of Rrp4 [29]. Given the high conservation of the S1 domain sequence in archaeal exosomes, we examined its potential applicability in *T. aci*. The pore created by the three S1 domains had a width of approximately 11 Å, allowing enough room for the single-stranded RNA to pass through, but not enough for the double-stranded RNA (Figure 14a). The electrostatic surface model of the S1 pore confirms that this pore functions as the route for RNA entry (Figure 14b).

**Figure 15:**
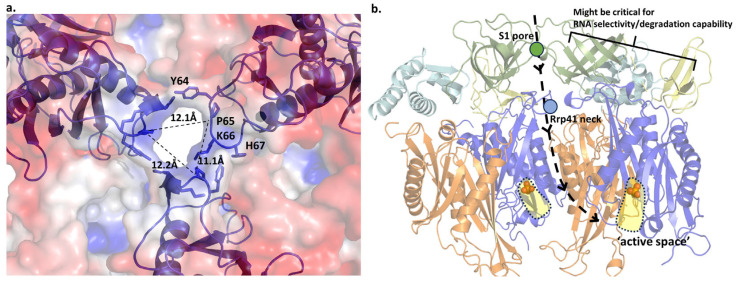
(**a**)The Rrp41 neck of *T. acidophilum.* The channel, formed of four residues per domain, is approximately 12 Å wide. (**b**) A schematic diagram depicting the pathway of ssRNA. For convenience, only six out of the nine subunits are depicted. The active space is represented in yellow color.

Once the ssRNA has traversed the S1 pore, it must also navigate through the constricted region created by Rrp41, known as the ‘neck’ [30]. The neck in *T. aci* consisted of four residues per domain: Y64, P65, K66, and H67 (Figure 15a). The neck created by these residues had a diameter of roughly 12 Å. This value was also adequate to differentiate between single-strand and double-strand substrates. Additionally, the neck, with a positive charge, seemed to enhance the ability to reach nucleic acid easily. The narrow diameters of these two entrances appeared to have substantial roles in determining substrate choice. Moreover, these confining environments were deemed to not only hinder the entry of secondary-structured RNA, but also to establish circumstances for the sequential degradation of individual substrates [31].

**Figure 16:**
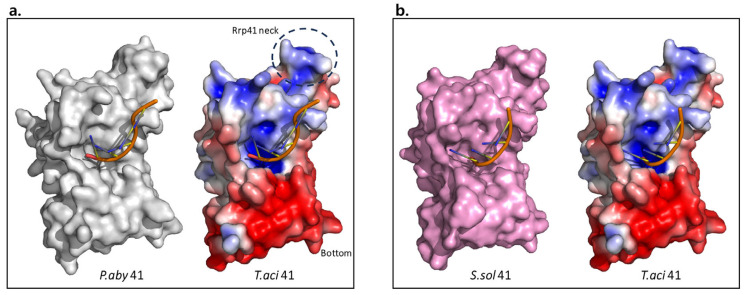
A view of the modeled RNA substrate from the PH-pore side of the RNase PH ring of Rrp41. (**a**) The *Taci*Rrp41 superimposed with the RNA-bound structure of the *Paby*Rrp41-RNA-bound structure (PDB ID: 2PO1). (**b**) *Taci*Rrp41 superimposed with the RNA-bound structure of *Ssol*Rrp41 (PDB ID: 2JEA). *Taci*Rrp41 is represented as an electrostatic surface potential. The range of electrostatic surface potential is shown from −5 kT/e (red color) to +5 kT/e (blue color).

In conclusion, ssRNA passes through two confined openings, namely the S1 pore and Rrp41 neck, to access the ‘active space’. Based on the position of phosphate found in the structure of *T. aci*, we could suggest a rough outline of the active space (Figure 15b). Upon comparison with other exosomes, it is evident that the active region is situated close to the interface between *Taci*Rrp41 and *Taci*Rrp42, with greater proximity to *Taci*Rrp41. The structural findings of *T. acidophilum* suggest that the exosomal phosphate-binding site is highly conserved among archaea. Based on the structure of the RNase PH ring complexed with single-stranded poly(A)-RNA of *P. aby* and *S. sol*, we modeled the RNA-bound structure with *Taci*Rrp41 by superimposing the structures (Figure 16a,b). In both cases, RNA was definitively located in the ‘active space’ of *Taci*Rrp41, the same region where inorganic phosphate electron density was found.

Given the significant conservation observed in the RNA substrate pathway, including the S1 pore, the 41neck, and the surrounding area of the active site, it is suggested that RNA-degrading ability has been evolutionarily conserved across species. Nevertheless, polymorphisms in other locations are likely to give rise to differences in RNA-degrading ability and substrate selectivity. We hypothesize that the most significant disparities between species would stem from Rrp4 due to two specific factors. First, each Rrp4 demonstrated the lowest degree of similarity when comparing archaeal exosomes. Rrp4’s possession of two RNA-binding motifs, S1 and KH, suggests that it may employ various ways to recognize or select RNA. Secondly, the distinct composition of the exosome, which is mainly influenced by the N-terminal domain of Rrp4, may lead to variations in RNA degradation as a result of the exosome’s capacity to bind, facilitated by the N-terminal domain.

## Figures and Tables

**Table 1:** Data collection and refinement statistics of the crystal structure from *Thermoplasma acidophilum* exosome.

	Rrp41:42	Rrp4:41:42
Data Collection		
Wavelength	1.000	1.000
Space group	P2_1_3	P3_2_21
a, b, c (A)	164.6, 164.6, 164.6	240.82, 240.82, 216.83
α, β, γ (°)	90, 90, 90	90, 90, 120
Resolution (A)	29.1–2.3 (2.4–2.3)	49.4–3.5 (3.6–3.5)
Unique reflections	65425 (6388)	86210 (7510)
Completeness	99.2 (98.4)	94.4 (83.2)
Multiplicity	5.4 (3.8)	4.1 (2.3)
I/σ(I)	23.1 (2.4)	5.3 (2.0)
Rmerge (%)	0.09 (0.45)	0.18 (0.39)
**Refinement Statistics**		
Resolution (A)	29.1–2.3	49.4–3.5
Reflections used in refinement	65413	86202
Rwork/Rfree	0.2042/0.2355	0.2302/0.2740
R.M.S deviations		
Bond lengths (A)	0.003	0.017
Bond angles (°)	0.86	1.77
Ramachandran plot (%)		
Favored	99.67	98.74
Allowed	0.33	1.16
No. atoms		
Protein	7105	16049
Ligands	20	0
Solvent	285	134
B-factors		
Protein	52.5	86.9
Ligands	64.1	0
solvent	49.9	49.4

Statistics for the highest-resolution shell are shown in parentheses.

## Data Availability

The accession numbers for the structures of *Taci*Rrp41/cd-*Taci*Rrp42 and *Taci*Rrp4/*Taci*Rrp41/cd-*Taci*Rrp42, as described in this paper, are PDB8XIE and PDB8XFX, respectively. All other data are included in this paper.
